# Supplementary material for: ITGB6-Knockout Suppresses Cholangiocarcinoma Cell Migration and Invasion with Declining PODXL2 Expression
Source: Int J Mol Sci. 2021 Jun 11;22(12):6303. doi: 10.3390/ijms22126303 (PMC8231266; doi:10.3390/ijms22126303)
Supplement: Supplementary file 1 [file ijms-22-06303-s001.zip › ijms-1202883-supplementary.pdf]

Table S1. Gene ontology and pathway analysis of downregulated genes in *ITGB6*-ko cell.

GO ID	GO Term	Count	Percent
GO:0007165	signal transduction	26	16.4%
GO:0055114	oxidation-reduction process	21	13.2%
GO:0007596	blood coagulation	10	6.3%
GO:0007267	**cell-cell signaling**	8	5.0%
GO:0001666	response to hypoxia	8	5.0%
GO:0030198	**extracellular matrix organization**	7	4.4%
GO:0010951	negative regulation of endopeptidase activity	5	3.1%
GO:0044267	cellular protein metabolic process	5	3.1%
GO:0034765	regulation of ion transmembrane transport	5	3.1%
GO:0006805	xenobiotic metabolic process	5	3.1%
GO:0030855	**epithelial cell differentiation**	4	2.5%
GO:0006906	vesicle fusion	4	2.5%
GO:0048791	calcium ion-regulated exocytosis of neurotransmitter	4	2.5%
GO:0017158	regulation of calcium ion-dependent exocytosis	4	2.5%
GO:0030203	glycosaminoglycan metabolic process	4	2.5%
GO:0009755	hormone-mediated signaling pathway	4	2.5%
GO:0048678	response to axon injury	4	2.5%
GO:0015671	oxygen transport	4	2.5%
KEGG pathway			
hsa01130	Biosynthesis of antibiotics	7	17.5%
hsa04145	Phagosome	6	15.0%
hsa04728	Dopaminergic synapse	6	15.0%
hsa04713	Circadian entrainment	5	12.5%
hsa04727	GABAergic synapse	5	12.5%
hsa01230	Biosynthesis of amino acids	4	10.0%
hsa05031	Amphetamine addiction	4	10.0%
hsa00630	Glyoxylate and dicarboxylate metabolism	3	7.5%

Table S2. Relationship between integrin β6 expression and clinicopathological characteristics of intrahepatic cholangiocarcinoma.

		Number of cases (n = 52)	β6 integrin expression		
			Negative (n = 21)	Positive (n = 31)	*p*-Value
Gender	Male	39	15	24	0.431
	Female	13	6	7	
Age (mean) (years)			73.4 (53-84)	69.1 (39-84)	0.114
Tumor size (mean) (mm)			59.1 (20-220)	58.7 (18-140)	0.975
Localization	Peripheral	41	19	22	0.0869
	Non-peripheral	11	2	9	
Macroscopic type	MF	46	21	25	0.0362*
	MF+PI, IG+PI, PI	6	0	6	
Histological differentiation	Well	7	3	4	0.170
	Moderate	32	10	22	
	Poor	13	8	5	
Growth type	Expansive	24	13	11	0.0555
	Infiltrative	28	8	20	
Serosa invasion	+	25	9	16	0.368
	-	27	12	15	
Portal vein invasion	+	42	15	27	0.148
	-	10	6	4	
Hepatic vein invasion	+	22	6	16	0.0856
	-	30	15	15	
Hepatic artery invasion	+	5	2	3	0.680
	-	47	19	28	
Bile duct invasion	+	27	8	19	0.0868
	-	25	13	12	
Intrahepatic metastasis	+	21	5	16	0.0417*
	-	31	16	15	
Lymph node metastasis	+	14	2	12	0.0194*
	-	38	19	19	

MF: mass-forming type, PI: periductal-infiltrating type, IG: intraductal-growth type; *, $p < 0.05$

Table S3. Primer sequences for PCR amplification after transfection.

Target DNA		Sequence
#1	Forward	AGCTATTAGATCCATTAA
	Reverse	TGACGTAGAGCATTTTAA
#2	Forward	GGATCTTGACCACACAGTTA
	Reverse	CATTCTGGTTTGCCACATC
#3	Forward	TTTCCTGTTGGGCTTTGCAG
	Reverse	GAAAGTGTCTGAGAGGCTGGGG

Table S4. Primer sequences for PCR amplification of expressed genes.

Gene name		Sequence
PODXL2	Forward	GCTGAAGCTCAATCCAGGATAC
	Reverse	CGGAACACCTCACAGTCTATGTTC
CLDN2	Forward	GGCTCCAGTGGGTGTTTCTA
	Reverse	GCTTGGTGCTATGGTCTTTCA
S100A2	Forward	AGCTTTGTGGGGGAGAAAGT
	Reverse	CAGTGATGAGTGCCAGGAAA
TSPAN8	Forward	TCTATGGTCCTGTATTGCCAGATC
	Reverse	GACAGCTGCTCCTGACTTATATAGC
LGALS1	Forward	CTCCTGACGCTAAGAGCTTCG
	Reverse	CCAGGCTGGAAGGGAAAGAC
CEACAM6	Forward	GGGTATCGCTGAGACTAAGTTGTA
	Reverse	CCTTAGGCAAGATACAAACCAAC
GAPDH	Forward	AACGGATTTGGTCGTATTGGG
	Reverse	CCTGGAAGATGGTGATGGGAT

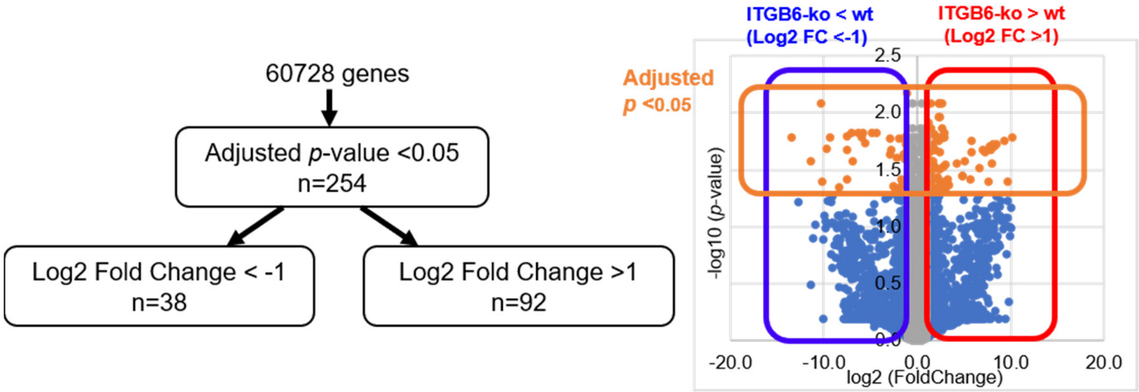

Figure S1. RNA sequencing analysis of HuCCT1 wild-type (wt) and integrin β6 knockout (*ITGB6*-ko) 1 cells. The differentially expressed genes in HuCCT1-wt and *ITGB6*-ko 1 cells were extracted by adjusted *p*-value and Log2 fold change, as shown in the volcano plot. We focused on the downregulated genes in *ITGB6*-ko 1 cells (n = 38). The P-value was adjusted using the Benjamini-Hochberg procedure.
